# Metal–Organic Framework‐Derived ZnO/ZnS Heteronanostructures for Efficient Visible‐Light‐Driven Photocatalytic Hydrogen Production

**DOI:** 10.1002/advs.201700590

**Published:** 2018-01-03

**Authors:** Xiuxia Zhao, Jianrui Feng, Jingwei Liu, Jia Lu, Wei Shi, Guangming Yang, Guichang Wang, Pingyun Feng, Peng Cheng

**Affiliations:** ^1^ College of Chemistry and Key Laboratory of Advanced Energy Materials Chemistry (MOE) Nankai University Tianjin 300071 China; ^2^ Collaborative Innovation Center of Chemical Science and Engineering (Tianjin) Tianjin 300071 China; ^3^ State Key Laboratory of Elemento‐Organic Chemistry Nankai University Tianjin 300071 China; ^4^ Department of Chemistry University of California Riverside CA 92521 USA

**Keywords:** hydrogen production, metal–organic frameworks, photocatalysts, ZnO/ZnS heteronanostructures

## Abstract

Developing highly active, recyclable, and inexpensive photocatalysts for hydrogen evolution reaction (HER) under visible light is significant for the direct conversion of solar energy into chemical fuels for various green energy applications. For such applications, it is very challenging but vitally important for a photocatalyst to simultaneously enhance the visible‐light absorption and suppress photogenerated electron–hole recombination, while also to maintain high stability and recyclability. Herein, a metal–organic framework (MOF)‐templated strategy has been developed to prepare heterostructured nanocatalysts with superior photocatalytic HER activity. Very uniquely, the synthesized photocatalytic materials can be recycled easily after use to restore the initial photocatalytic activity. It is shown that by controlling the calcination temperature and time with MOF‐5 as a host and guest thioacetamide as a sulfur source, the chemical compositions of the formed heterojunctions of ZnO/ZnS can be tuned to further enhance the visible‐light absorption and photocatalytic activity. The nanoscale heterojunction ZnO/ZnS structural feature serves to reduce the average free path of charge carriers and improve the charge separation efficiency, thus leading to significantly enhanced HER activity under visible‐light irradiation (λ > 420 nm) with high stability and recyclability without any cocatalyst.

## 1 Introduction

Hydrogen, as a clean and renewable energy carrier, has attracted tremendous attention because of its great potential to replace traditional fossil fuels.^1^, ^2^ Hydrogen evolution by photocatalytic water splitting represents one of the most promising technologies due to the use of renewable and clean solar energy.^3^, ^4^, ^5^, ^6^ As a photocatalyst for hydrogen evolution, its efficiency greatly depends on the catalyst's capabilities of absorbing visible light and suppressing photogenerated electron–hole recombination.^7^ Compared with a single catalyst, heterostructured nanomaterials or nanocomposites could combine the strengths of each individual material and solve abovementioned problems.^8^, ^9^, ^10^ Fundamentally, the nanosized structural feature could cause a significant shift in the position of the band edge of the material to achieve the absorption of visible light^11^, ^12^ and provide higher surface area so as to potentially increase the accessible reaction sites for the photocatalysis.^13^ In recent years, numerous efforts have been devoted to the design and fabrication of heterojunction photocatalysts for tailoring band gaps and improving the photocatalytic activity.^14^, ^15^ Two most‐used synthesis methods, interfacing different materials via aggregation,^16^ and epitaxial nucleation of one material on the surface of the other,^17^ have made a big contribution to this field but tend to lower the accessible active surface area and reduce the number of active sites. Therefore, it is essential to exploit new methods for the synthesis of heterojunctions, especially nanosized heterojunctions, for photocatalytic hydrogen evolution.

The ZnO/ZnS heterostructures are promising photocatalysts for hydrogen evolution because ZnO has photogenerated holes with high oxidizing power^18^ and ZnS possesses both rapid generation of electron–hole pairs by photoexcitation and the highly negative reduction potential of photoexcited electrons.^19^ More importantly, the bottom of the conduction band of ZnO is located in the band gap of ZnS,^20^ yielding a superior material with a lower photoexcitation threshold than that of individual components.^21^ Especially in nanometer‐scaled structures, the spatial proximity of the band edge wave functions to the interface would lead to an increase in the oscillator strength, which plays a decisive role in obtaining the material with small band gap and a visible‐light response from two materials with large band gap.^22^ In addition, ZnO and ZnS are low cost and environmentally benign, and have high electrochemical stability and electron mobility that are indispensable for the efficient photocatalysts toward water splitting reaction.^23^, ^24^ So far, ZnO/ZnS composites including heterojunction particles and core/shell nanorods have been fabricated for photocatalytic application by the sulfidization or oxidation process.^25^, ^26^, ^27^ These materials are usually large in particle size or synthesized by coating one component over the other. The large size leads to a long migration distance for the carriers to the photocatalyst surface, resulting in a low charge‐carrier separation efficiency.^9^ The coating typically leads to a configuration in which the holes or electrons by photoexcitation gather in the inner component of the core/shell nanorod, making it difficult to migrate out. This could also hinder the separation of electrons and holes and limit the photocatalytic efficiency. Therefore, fabricating ZnO/ZnS nanosized heterojunctions for photocatalytic hydrogen evolution is still imperative and challenging.

Metal–organic frameworks (MOFs) are crystalline open‐framework materials with tunable pore structures and have shown great potentials in many fields.^28^, ^29^, ^30^, ^31^, ^32^ Recently, the use of MOFs as precursor for the synthesis of new materials is undergoing the rapid development because of the compositional and structural diversities of MOFs. Such diverse features provide new opportunities to efficiently integrate MOF materials with other chemicals and to further tune the materials with desired properties.^33^, ^34^, ^35^, ^36^, ^37^ More importantly, taking advantage of long‐range ordering at a molecular level, MOFs have been demonstrated to be highly useful precursors and templates for the synthesis of nanostructured carbon,^38^ carbon decorated MO quantum dots,^39^ and nanocomposites M/MO@C (M = metal, MO = metal oxide).^40^

**Figure 1:**
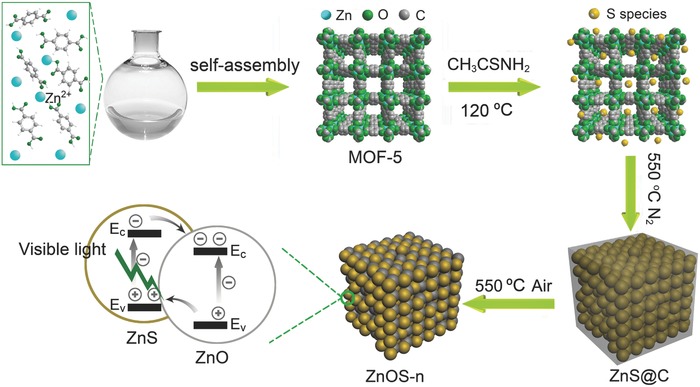
Schematic illustration of the synthetic procedure of ZnO/ZnS heterostructures.

In this contribution, we report a novel and facile method to prepare ZnO/ZnS nanostructures with an average size of 20 nm and uniform composition distribution. These nanostructures were synthesized via solvothermal synthesis followed by high temperature treatments, in which H_2_S was produced from the alcoholysis of thioacetamide (TAA) and subsequently used in a high temperature treatment for the transformation of MOF‐5 to ZnS@C. The formed ZnS@C was further calcined in air to produce the nanostructured ZnO/ZnS materials (**Figure** **1**). Importantly, by adjusting the calcination time of ZnS@C in air, a family of ZnO/ZnS nanostructured materials with different ZnO/ZnS ratios was obtained. They are named as ZnOS‐n: ZnOS‐15, ZnOS‐30, ZnOS‐45, and ZnOS‐60 (here n represents different treatment times in air leading to different ZnO/ZnS ratios). Among these synthesized ZnOS‐n materials, ZnOS‐30 shows excellent photocatalytic activity (415 µmol h^−1^ g^−1^) under visible light, which is one of the best photocatalysts for hydrogen evolution reaction (HER). Moreover, ZnOS‐30 demonstrates high stability and excellent recyclability, which could attribute to the synergetic effect of heterostructural feature of ZnO and ZnS nanostructures.

## 2 Results and Discussion

### 2.1 Characterization

**Figure 2:**
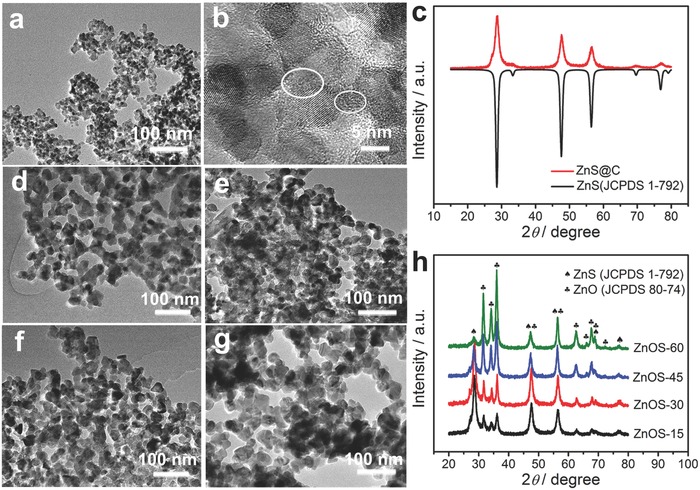
a) TEM image, b) HRTEM image, c) PXRD of ZnS@C. TEM images of d) ZnOS‐15, e) ZnOS‐30, f) ZnOS‐45, and g) ZnOS‐60. h) PXRD patterns of ZnOS‐n.

A new reflux method rather than conventional hydrothermal method^41^ was used for large‐scale preparation of the microsized particles of MOF‐5 (Figures S1–S3, Supporting Information and Experimental Section), which is facile and economic. During the hydrothermal treatment of the as‐synthesized MOF‐5 in TAA ethanol solution, the ethanol could be added to the C=S bond to give CH_3_(NH_2_)C(OC_2_H_5_)‐SH. This process could result in the formation of CH_3_(NH_2_)C(OC_2_H_5_)_2_ and H_2_S.^37^ The released H_2_S diffused into the channels of MOF‐5 and reacted with MOF‐5 to yield the rough ZnS particles, which were characterized by powder X‐ray diffraction (PXRD) and scanning electron microscopy (Figures S4 and S5, Supporting Information). Upon further thermal annealing in N_2_ atmosphere, the light‐yellow compound was converted into the carbon‐encapsulated highly crystalline ZnS nanoparticles (**Figure** **2**a–c). Finally, ZnS@C was calcined in air to obtain ZnO/ZnS heterostructures (Figure 2d–h). The temperature of 550 °C was chosen for carbonization of the rough ZnS under optimized N_2_ velocity. The obtained samples were investigated by transmission electron microscopy (TEM) and PXRD. Zinc sulfide nanoparticles (≈7 nm, Figure S6, Supporting Information) are surrounded by uniform porous carbon matrices (Figure 2a–c). All the diffraction peaks matched well with the sphalerite zinc sulfide (JCPDS 1–792). The second calcination process was conducted in air at 450, 550, and 700 °C for 30 min (Figure S7, Supporting Information). The results showed that as calcining temperature increased the colors of the samples gradually shallow from black to white (Figure S7 inset, Supporting Information), indicating that increasing calcining temperature in air is conducive to the removal of carbon residue. Simultaneously, the chemical composition changed from ZnS@C to ZnO/ZnS and finally to ZnO (Figure S8, Supporting Information). In order to partly transform ZnS to ZnO, ZnS@C nanoparticles were calcined in air at 550 °C for the formation of ZnO/ZnS heterostructures. The resulting ZnO/ZnS heterostructures were characterized by high‐resolution TEM (HRTEM) and PXRD (Figure 2d–h). The HRTEM images reveal that ZnO/ZnS heterostructures are nanoparticles with size of ≈20 nm. All the diffraction peaks of PXRD can be assigned to ZnO (JCPDS card no. 80–74) and ZnS (JCPDS card no. 1–792). The ratios of ZnO and ZnS can be controlled by modulating the calcination time of ZnS@C composite in air for 15, 30, 45, and 60 min, and the finally obtained ZnO/ZnS heterostructures were denoted as ZnOS‐15, ZnOS‐30, ZnOS‐45, and ZnOS‐60, respectively. As shown in Table S1 (Supporting Information), the content of ZnS in ZnOS‐n decreases with the increasing annealing time in air. The compositions of ZnOS‐n are determined by X‐ray photoelectron spectroscopy (XPS), thermogravimetry analysis (TGA, Figure S9, Supporting Information) in air, and energy dispersive X‐ray spectroscopy (EDS, Figure S10, Supporting Information).

**Figure 3:**
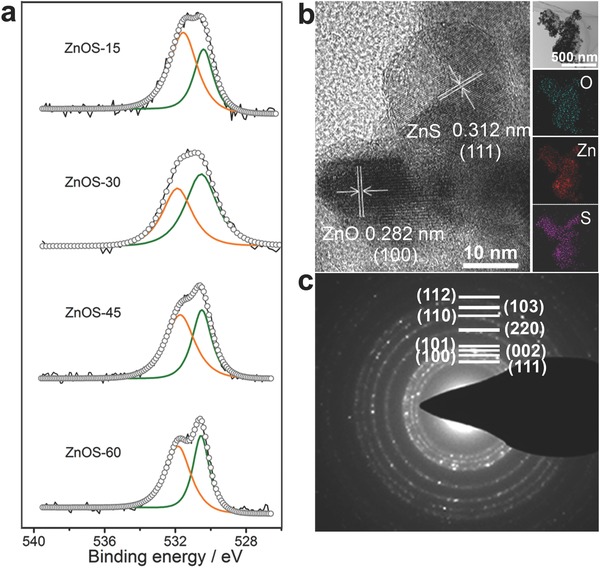
a) XPS survey spectra with the corresponding O 1s spectra of ZnOS‐n. b) HRTEM image, EDS spectra (panel b inset), c) SAED of ZnOS‐30.

In order to further investigate the surface composition of ZnOS‐n, XPS survey spectra with the corresponding O 1s, S 2p, Zn 2p, and C 1s spectra were studied. As shown in **Figure** **3**a, the O 1s peak in ZnO/ZnS heterostructures is well divided into two peaks at 530.5 and 531.8 eV. The lower energy peak at 530.5 eV indicates the formation of ZnO,^42^ and the higher energy peak at 531.8 eV is usually ascribed to chemisorbed oxygen on the surface of ZnO/ZnS nanoparticles.^43^ The position of the S 2p peak (Figure S11, Supporting Information), Zn 2p_3/2_, and Zn 2p_5/2_ peak (Figure S12, Supporting Information) can be observed at 162.4, 1021.9, and 1044.9 eV, respectively, which suggested that S and Zn elements exist mainly in the form of S^2−^ and Zn^2+^ on the surface of the samples.^24^ The presence of weak C 1s peak further confirms the residual small amount carbon in ZnO/ZnS heterostructures (Figure S13, Supporting Information).

As an efficient photocatalyst, a high charge‐carrier separation is of crucial importance in photocatalytic reactions. From the high‐magnification HRTEM image (Figure 3b), the sizes of ZnOS‐n particles are all in the range of 16–24 nm (Figure S14, Supporting Information), which imply that carriers have short distances to migrate to the surface and can be beneficial for improving the charge‐carrier separation efficiency.^44^ The pore size distributions showed that the pores are in the range of 9–13 nm for ZnOS‐n (Figure S15, Supporting Information). Besides, HRTEM shows that ZnO/ZnS heterostructures have abundant coupling interface between ZnO and ZnS nanoparticles, which could greatly enhance the electron transport and promote the photochemical activity. For a typical nanoparticle, the clear lattice spacing of 0.282 and 0.312 nm can be indexed to the (100) plane of ZnO and the (111) plane of ZnS, respectively. It is noted that there should be a mismatch between these two types of the nanostructures of ZnO and ZnS at the boundary.^45^, ^46^ Elemental mapping of ZnOS‐30 was performed to further confirm that Zn, O, and S elements are evenly distributed on the nanoparticles and suggest that the species of ZnO and ZnS are connected to each other in nanoscale rather than isolated (Figure 3b inset). In addition, the clear fringes observed in the selected area electron diffraction (SAED, Figure 3c) indicate the polycrystalline property of ZnO/ZnS nanoparticles and presence of (‐101) (100) (002) (110) (103) planes of ZnO (JCPDS card no. 80–74) and (‐111) (220) (311) planes of ZnS (JCPDS card no. 1–792).

**Figure 4:**
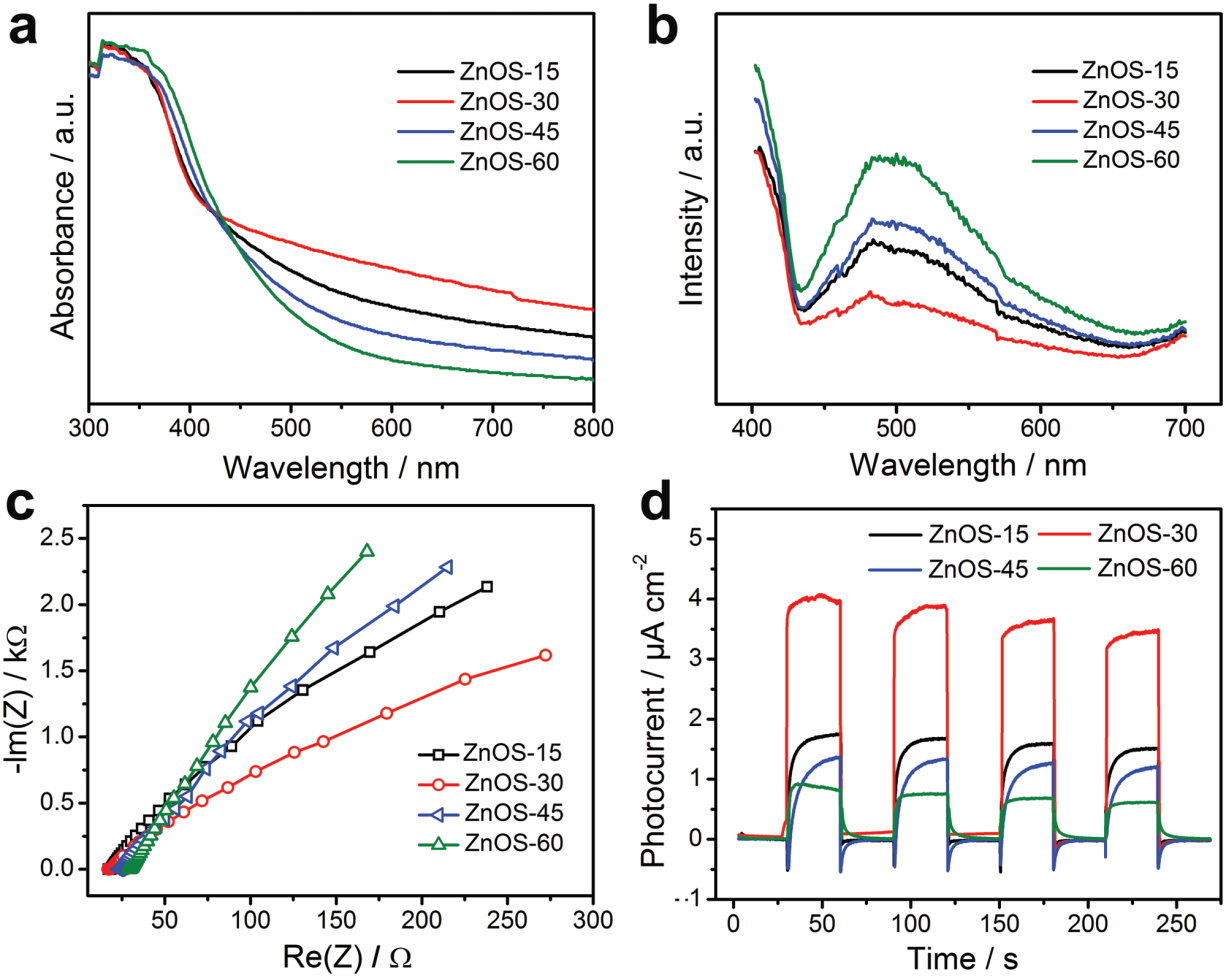
a) UV–vis absorption spectra, b) PL emission spectra (excitation wavelength = 360 nm), c) EIS plots, and d) photocurrent‐time dependence of ZnOS‐n.

The UV–vis diffuse reflectance spectra of ZnOS‐n were measured to confirm their light‐harvesting abilities. As shown in **Figure** **4**a, increasing the concentrations of ZnO in ZnO/ZnS heterostructures led to improved intensities of visible‐light absorption from ZnOS‐15 to ZnOS‐30 and decreased intensities from ZnOS‐30 to ZnOS‐60. The minimal residual carbon was confirmed by element analysis (EA, Table S2, Supporting Information). The gradual decrease of the carbon content from ZnOS‐15 to ZnOS‐60 could reduce the light absorption capacity of full spectrum gradually. However, the order of light‐harvesting abilities as shown in Figure 4a demonstrates that the light‐harvesting abilities of ZnO/ZnS heterostructures are not dominated by the trace amount of conductive carbon. In addition, ZnS@C and the nanoparticles obtained at 450 °C in air with high carbon content showed strong visible‐light absorption capability (Figure S16, Supporting Information) but negligible photocatalytic HER activity under visible‐light irradiation (Figure S17, Supporting Information), further indicating that the photocatalytic property is from the ZnO/ZnS heterostructure. The optimal visible‐light absorption of the synthesized ZnO/ZnS nanostructures is found with ZnOS‐30.

To investigate the charge‐carrier separation/recombination rates of the photoexcited carriers, photoluminescence (PL) spectra under excitation wavelength of 360 nm were performed (Figure 4b). The results show that PL intensity of ZnOS‐n decreases first and then increases subsequently with increasing concentration of ZnO in ZnO/ZnS heterostructures, among which ZnOS‐30 exhibits the weakest PL intensity. The PL quenching behavior suggests the effective suppression of the electron–hole recombination,^47^ while the lower recombination rate can facilitate the heterogeneous photocatalysis. In accordance with the PL spectra, ZnOS‐30 exhibits the smallest Nyquist plot diameter in the electrochemical impedance spectra (EIS, Figure 4c), indicating its low charge‐transfer resistance.^48^, ^49^, ^50^ Moreover, the photocurrent density of ZnOS‐30 is the highest in the ZnOS‐n (Figure 4d), illustrating its high efficient separation of photoexcited charge carriers,^51^ which is attributed to its abundant coupling interface between ZnO and ZnS nanoparticles resulting from the almost equal amounts of ZnO and ZnS in ZnOS‐30.

**Figure 5:**
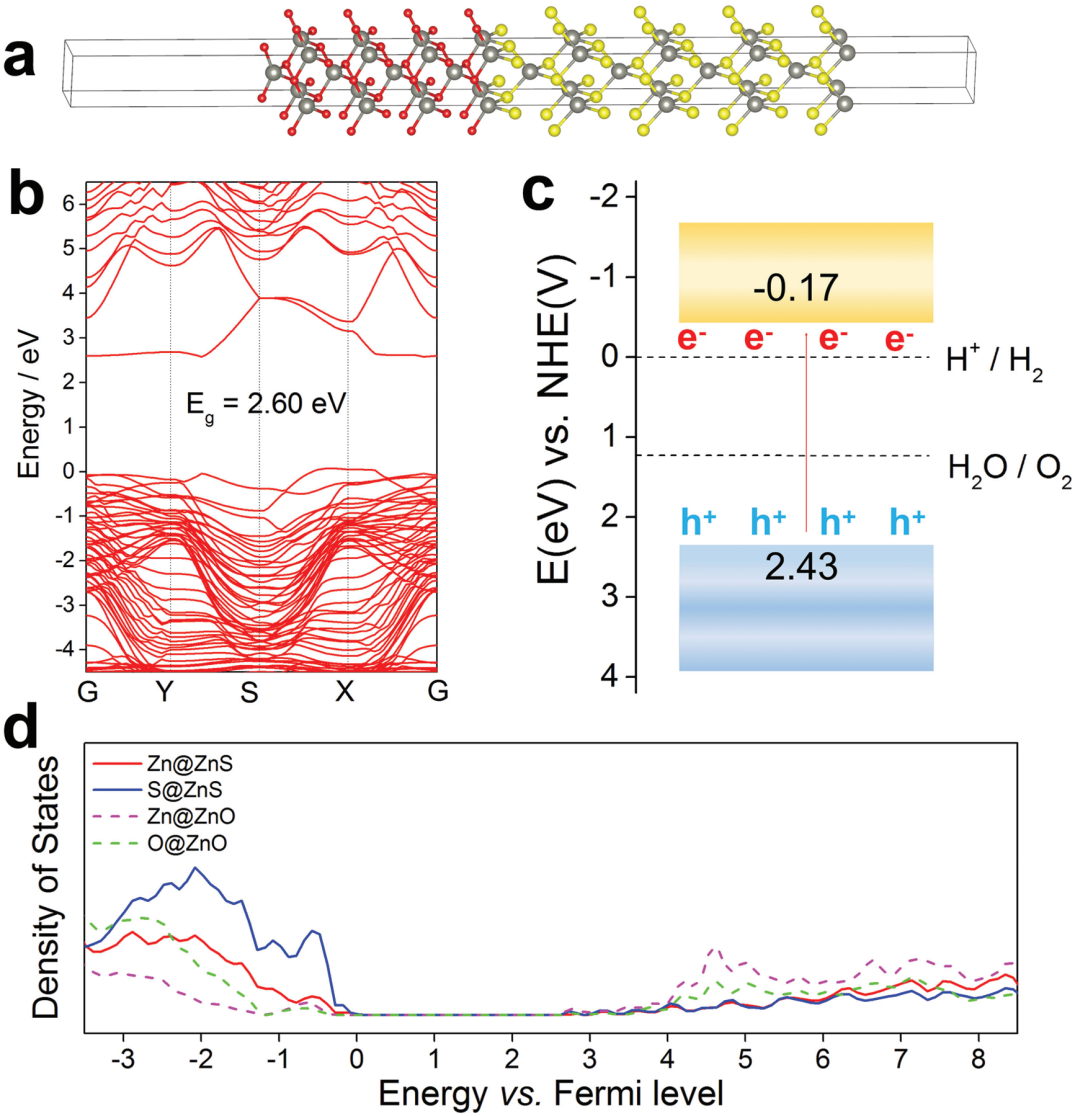
The first‐principle calculations on the electronic structure of ZnO/ZnS heterostructures. a) Optimized geometric structure of ZnO/ZnS heterojunction interface. Red, yellow, and gray balls denote O, S, and Zn atoms, respectively. b) Calculated band structure plots of ZnO/ZnS interface. c) Schematic illustration of band alignment and carrier separation in the proposed heterostructure. d) Projected density of states of ZnO/ZnS heterojunction.

To understand the fundamental electronic structure of ZnO/ZnS heterostructure, first‐principle calculations were performed on the basis of hybrid density functional theory (DFT) method. Optimized ZnO(001)/ZnS(001) heterostructure slab model was adopted as **Figure** **5**a. In this model, a mutual Zn atom was shared by two phases as the interface of heterostructure. The band structures are depicted in Figure S18 (Supporting Information) and Figure 5b. It is clearly seen that the ZnO/ZnS heterojunction presents a semiconductor character with an indirect band gap of 2.60 eV from ZnO (3.26 eV) and ZnS (3.72 eV), which is consistent with the result of UV–vis diffuse reflectance spectra in Figure 4a. As shown in the band structure, valence band maximum (VBM) locates between S and X point and conduction band minimum is between Y and S point. The band alignment is illustrated in Figure 5c. The conduction band minimum (CBM) edge position of ZnO/ZnS locates at −0.17 V, which is more negative than that of H^+^/H_2_ (0 V). In this case, electrons are excited by light irradiation and transferred to H^+^, and hence could reduce H^+^ to H_2_. The projected density of states of ZnO/ZnS heterojunction (Figure 5d) indicates that VBM and CBM are derived from ZnS and ZnO, respectively. Schrier et al. have calculated that the band gap of ZnO/ZnS core/shell nanowires can be 2.07 eV,^22^ which is close to our calculation result and further lays the theoretical foundation for ZnO/ZnS heterostructures as photocatalysts under visible‐light irradiation.

### 2.2 Photocatalytic Performance for Hydrogen Evolution Reaction

**Figure 6:**
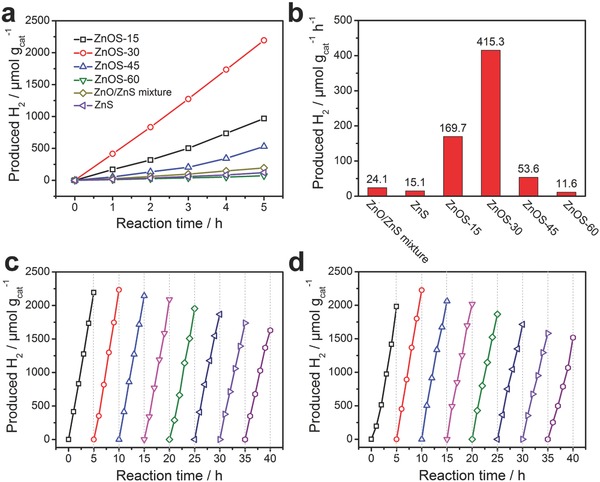
Photocatalytic performance of ZnO/ZnS heterostructures. a) Time‐dependent photocatalytic hydrogen evolution under visible‐light irradiation (λ > 420 nm, 300 W Xe lamp) for ZnOS‐n. b) Comparison of photocatalytic HER of ZnS/ZnO mixture, ZnS and ZnOS‐n under visible‐light irradiation. c) Cycling test of H_2_ evolution (evacuation every 5 h) for ZnOS‐30 under visible‐light irradiation. d) Cycling test of H_2_ evolution (evacuation every 5 h) for recycled and reprocessed ZnOS‐30 after 40 h cycling test.

The work demonstrated that the ZnO/ZnS heterostructure is a promising photocatalyst for reducing water to form H_2_ under visible‐light irradiation. We further investigated the photocatalytic activity of the synthesized materials for the production of hydrogen in the presence Na_2_S and Na_2_SO_3_ as a sacrificial electron donor without the use of any cocatalysts under visible light (>420 nm, **Figure** **6**a). As shown in Figure 6b, with the increasing of the amount of ZnO in ZnOS‐n, HER gradually speeds up and reaches an optimum as high as 435 µmol g^−1^ h^−1^ by ZnOS‐30. The HER rate then decreases upon further increasing the ZnO content. Notably, the relative content of ZnO and ZnS in composite ZnOS‐n played an important role for photocatalytic H_2_ production activity. When ZnS@C was calcined in air for 15 and 60 min, ZnOS‐15 and ZnOS‐60 formed with the minimum and the maximum content of ZnO, respectively. In composite ZnOS‐15, ZnO as the gathering place of photoexcited electron may not be enough on the surface, resulting in the decreased photocatalytic activity. On the contrary, in composite ZnOS‐60, the ZnS is covered by ZnO and photoexcited holes on the ZnS will not be able to contact with the hole scavengers efficiently.^52^ The high photocatalytic activity of ZnOS‐30 in comparison with other ZnOS‐n (*n* = 15, 45, and 60) may result from the abundant coupling interface between ZnO and ZnS nanoparticles, which improves interfacial charge transfer and therefore effective charge separation.^53^, ^54^ In addition, the HER catalytic results match well with the intensities of photocurrent of the materials. For comparison, ZnS nanoparticles and the mixture of ZnO and ZnS nanoparticles by milling were also tested under the same reaction conditions, and both exhibited much low visible‐light HER activities.

The stability of ZnOS‐30 was evaluated by carrying out photocatalytic hydrogen evolution under identical reaction conditions for eight cycles. As shown in Figure 6c, even after 40 h of light irradiation, 75% of the original catalytic activity was maintained. The reason for the reduced photocatalytic activity is that ZnO in ZnO/ZnS heterostructures gradually transformed into ZnS in Na_2_S and Na_2_SO_3_ solution under irradiation. It is noteworthy that the recycled ZnOS‐30 could restore the original photocatalytic activity after reannealing again in air for 30 min (Figure 6d). This leads to the recovery of ZnS to ZnO/ZnS in ZnOS‐30, which is proved by PXRD (Figure S19, Supporting Information). Impressively, the performance of ZnOS‐30 is among the best reported congeneric photocatalysts with high visible‐light photocatalytic activity and photostability (Table S3, Supporting Information).

## 3 Conclusion

A new type of ZnO/ZnS nanostructures has been fabricated by coupling solvothermal synthesis with a two‐step calcination strategy for photocatalytic hydrogen production under visible light. In the heteronanostructures, ZnO and ZnS nanoparticles are homogeneously integrated owning to the uniform sulfidation of the MOF precursor at the molecular level and subsequent oxidization of the nanoscale ZnS@C. This new synthesis method significantly improves the interfacial catalytic active sites and makes it possible to optimize the light absorption capability and charge separation efficiency. In particular, the light‐harvesting ability can be well tuned by regulating the content of ZnO in the ZnO/ZnS heteronanostructures, leading to the optimized ZnOS‐30 that shows excellent photocatalytic HER activity and recoverability under visible light. The strategy reported here, i.e., homogeneous nanoscale ZnO/ZnS heterostructures from a MOF‐assisted route, provides a new approach to synthesize the uniformly integrated heteronanostructures of metal oxides and sulfides for catalysis and energy conversion.

## 4 Experimental Section

*Synthesis of MOF‐5*: Zn(NO_3_)_2_·6H_2_O (2.98 g, 10 mmol) was dissolved in 25 mL *N*,*N*‐dimethylmethanamide (DMF) to form a solution, which was subsequently poured into 65 mL DMF solution containing 1,4‐benzenedicarboxylate (0.33 g, 2 mmol). After thorough mixing, the solution was refluxed at 100 °C for 12 h. The resulting white precipitates were collected by centrifuging, washed with DMF and methanol in sequence for at least three times, and finally dried in vacuum at 70 °C overnight.

*Synthesis of ZnOS‐n*: 80 mg of the as‐prepared MOF‐5 and 160 mg of thioacetamide were added into a 23 mL a Teflon‐lined stainless‐steel autoclave, followed by the addition of 16 mL of absolute ethanol. After heating at 120 °C for 4 h, the mixture was centrifuged, washed with ethanol for three times, and dried at 75 °C overnight. Then, the product was annealed under N_2_ atmosphere at 550 °C for 2 h with the heating speed of 5 °C min^−1^ to obtain ZnS@C. Finally, the obtained ZnS@C was heat treated at target temperature (550 °C) for *n* min (*n* = 15, 30, 45, and 60) with the heating speed of 10 °C min^−1^ under air to obtain a series of products denoted ZnOS‐n. The compressed anhydrous air in cylinder was used as the air source for this thermal oxidation.

*Materials and Characterizations*: All the reagents in this experiment were analytical grade and used as received without further purification. PXRD was performed on a Rigaku Ultima IV diffractometer using Cu Kα radiation. TGA was performed in nitrogen flow on NETZSCH TG 209F3. Field‐emission scanning electron microscopy images were measured on JEOL JSM‐7500F scanning electron microscope. TEM images were taken on FEI Tecnai G2F20 electron microscope (200 kV). XPS were obtained using a PHI5000 VersaProbe XPS spectrometer. EA (C, H, and N) were characterized by Perkin‐Elmer 2400‐II CHNS/O analyzer. UV–vis spectra were performed on a Jasco V‐570 spectrophotometer.PL spectra were measured on an Agilent Cary Eclipse spectrophotometer.

*Computational Method*: Calculations were based on DFT. The Vienna ab initio simulation package^55^, ^56^ was implemented to optimize the structures and investigate their properties. To optimize geometric structures, ion–electron interactions were depicted by projector augmented waves^57^, ^58^ when the function of Perdew–Burke–Ernzerhof (PBE)^59^ based on the generalized gradient approximation was adopted to describe the exchange and correlation potential. Considering that the PBE function underestimates the band gaps of semiconductors in general, electronic structures are calculated using hybrid Hartree–Fock/DFT calculations^60^ with the mixing parameter of 0.5 and 0.5 for fraction of exact exchange and fraction of gradient correction to exchange. In this calculation, 5 × 5 × 1 and 15 × 15 × 1 Monkhorst–Pack^61^ sampled k points were used for geometry optimizations and electronic structure calculations, respectively. Cut‐off energy of 400 eV was adopted. The vacuum space is set to be at least 20 Å to separate the interactions between neighboring slabs. Criteria of convergence were set to 1 × 10^−4^ eV and 0.01 eV Å^−1^ for the self‐consistent field and ion steps, respectively.

*Photoelectrochemical Measurements*: Photoelectrochemical measurements were conducted with a CHI660D Electrochemical System in a conventional three electrode cell, using a Pt plate as the counterelectrode, and an Ag/AgCl electrode (0.1 m Na_2_SO_4_) as the reference electrode. The working electrode was prepared on indium‐tin oxide (ITO) glass that was cleaned by sonication in ethanol for 30 min and dried at 353 K. The boundary of ITO glass was protected using Scotch tape. The 10 mg catalyst was dispersed in 1 mL of ethanol by sonication for 1 h to get a slurry, then the slurry was spread onto pretreated ITO glass. After natural drying overnight, the working electrode was further dried at 393 K for 5 h to improve adhesion.

*General Procedure for Photocatalytic H_2_ Evolution*: The photocatalytic hydrogen evolution experiments were carried out under 300 W Xe lamp (PLS‐SXE 300C, Beijing PerfectLight Co. Ltd, China, λ > 420 nm), and the reactor was a top‐irradiation‐type Pyrex reaction cell connected to a closed gas circulation and evacuation system. The wavelength of the incident light was controlled by employing 420 nm long‐pass cut‐off filter. In a typical experiment, 50 mg catalyst was suspended in 100 mL aqueous solution, which contained Na_2_S (10.0 mmol, 2.4 g) and Na_2_SO_3_ (10.0 mmol, 1.26 g) as sacrificial electron donor. Afterward, the reaction system was sealed carefully before evacuated several times to remove air completely. Finally, the sealed quartz reactor was irradiated with the fixed distance (10 cm). During the photocatalytic reaction, the reaction solution was kept with a continuous magnetic stirring and 15 °C by a flow of cooling water. The evolved gases were analyzed by gas chromatography equipped with a thermal conductive detector and a 5 Å molecular sieve column, using N_2_ as the carrier gas.

## Conflict of Interest

The authors declare no conflict of interest.

## Supporting information

Supplementary

## References

[1] L. Schlapbach , A. Züttel , Nature 2001, 414, 353. 11713542 10.1038/35104634

[2] M. Ball , M. Wietschel , Int. J. Hydrogen Energy 2009, 34, 615.

[3] J. A. Turner , Science 2004, 305, 972. 15310892 10.1126/science.1103197

[4] S. Y. Tee , K. Y. Win , W. S. Teo , L.‐D. Koh , S. Liu , C. P. Teng , M.‐Y. Han , Adv. Sci. 2017, 4, 160037. 10.1002/advs.201600337 PMC5441509 28546906

[5] T. Hisatomi , J. Kubota , K. Domen , Chem. Soc. Rev. 2014, 43, 7520. 24413305 10.1039/c3cs60378d

[6] J. Thote , H. B. Aiyappa , A. Deshpande , D. D. Díaz , S. Kurungot , R. Banerjee , Chem. ‐ Eur. J. 2014, 20, 15961. 25307944 10.1002/chem.201403800

[7] X. B. Chen , L. Liu , P. Y. Yu , S. S. Mao , Science 2011, 331, 746. 21252313 10.1126/science.1200448

[8] D. V. Talapin , J.‐S. Lee , M. V. Kovalenko , E. V. Shevchenko , Chem. Rev. 2010, 110, 389. 19958036 10.1021/cr900137k

[9] H. L. Wang , L. S. Zhang , Z. G. Chen , J. Q. Hu , S. J. Li , Z. H. Wang , J. S. Liu , X. C. Wang , Chem. Soc. Rev. 2014, 43, 5234. 24841176 10.1039/c4cs00126e

[10] H. J. Li , W. G. Tu , Y. Zhou , Z. G. Zou , Adv. Sci. 2016, 3, 1500389. 10.1002/advs.201500389 PMC5102663 27980982

[11] L. Liao , Q. H. Zhang , Z. H. Su , Z. Z. Zhao , Y. N. Wang , Y. Li , X. X. Lu , D. G. Wei , G. Y. Feng , Q. K. Yu , X. J. Cai , J. M. Zhao , Z. F. Ren , H. Fang , F. Robles‐Hernandez , S. Baldelli , J. M. Bao , Nat. Nanotechnol. 2014, 9, 69. 24336404 10.1038/nnano.2013.272

[12] J. Jasieniak , M. Califano , S. E. Watkins , ACS Nano 2011, 5, 5888. 21662980 10.1021/nn201681s

[13] J. Zhang , Z. P. Zhu , Y. P. Tang , K. Müllen , X. L. Feng , Adv. Mater. 2014, 26, 734. 24249582 10.1002/adma.201303571

[14] Q. Xiang , J. Yu , M. Jaroniec , Chem. Soc. Rev. 2012, 41, 782. 21853184 10.1039/c1cs15172j

[15] K. Li , M. Han , R. Chen , S. L. Li , S. L. Xie , C. Y. Mao , X. H. Bu , X. L. Cao , L. Z Dong , P. Y. Feng , Y. Q. Lan , Adv. Mater. 2016, 28, 8906. 27553983 10.1002/adma.201601047

[16] Y. Sasaki , H. Nemoto , K. Saito , A. Kudo , J. Phys. Chem. C 2009, 113, 17536.

[17] K. Ma , O. Yehezkeli , D. W. Domaille , H. H. Funke , J. N. Cha , Angew. Chem., Int. Ed. 2015, 54, 11490. 10.1002/anie.201504155 26136433

[18] C. Hariharan , Appl. Catal., A 2006, 304, 55.

[19] D. G. Chen , F. Huang , G. Q. Ren , D. S. Li , M. Zheng , Y. Z. Wang , Z. Lin , Nanoscale 2010, 2, 2062. 20689875 10.1039/c0nr00171f

[20] D. Wu , Y. Jiang , Y. Yuan , J. Wu , K. Jiang , J. Nanopart. Res. 2011, 13, 2875.

[21] K. Zhang , D. W. Jing , Q. Y. Chen , L. J. Guo , Int. J. Hydrogen Energy 2010, 35, 2048.

[22] J. Schrier , D. O. Demchenko , L. Wang , Nano Lett. 2007, 7, 2377. 17645365 10.1021/nl071027k

[23] J. Zhang , J. Yu , Y. Zhang , Q. Li , J. R. Gong , Nano Lett. 2011, 11, 4774. 21981013 10.1021/nl202587b

[24] H. X. Sang , X. T. Wang , C. C. Fan , F. Wang , Int. J. Hydrogen Energy 2012, 37, 1348.

[25] W. N. Jia , B. X. Jia , H. M. Lin , F. Y. Qu , X. Wu , J. J. Jiang , Micro Nano Lett. 2011, 6, 633.

[26] A. J. Mieszawska , R. Jalilian , G. U. Sumanasekera , F. P. Zamborini , Small 2007, 3, 722. 17444570 10.1002/smll.200600727

[27] C. L. Yan , D. F. Xue , J. Phys. Chem. B 2006, 110, 25850. 17181231 10.1021/jp0659296

[28] J. R. Long , O. M. Yaghi , Chem. Soc. Rev. 2009, 38, 1213. 19384431 10.1039/b903811f

[29] H. C. Zhou , J. R. Long , O. M. Yaghi , Chem. Rev. 2012, 112, 673. 22280456 10.1021/cr300014x

[30] H. Furukawa , K. E. Cordova , M. O'Keeffe , O. M. Yaghi , Science 2013, 341, 1230444. 23990564 10.1126/science.1230444

[31] H. C. Zhou , S. Kitagawa , Chem. Soc. Rev. 2014, 43, 5415. 25011480 10.1039/c4cs90059f

[32] S. Saha , G. Das , J. Thote , R. Banerjee , J. Am. Chem. Soc. 2014, 136, 14845. 25279940 10.1021/ja509019k

[33] Y. K. Hwang , D.‐Y. Hong , J.‐S. Chang , S. H. Jhung , Y.‐K. Seo , J. Kim , A. Vimont , M. Daturi , C. Serre , G. Férey , Angew. Chem., Int. Ed. 2008, 47, 4144. 10.1002/anie.200705998 18435442

[34] J. Yu , Y. Cui , H. Xu , Y. Yang , Z. Wang , B. Chen , G. Qian , Nat. Commun. 2013, 4, 2719. 24173352 10.1038/ncomms3719 PMC4089137

[35] Q.‐L. Zhu , J. Li , Q. Xu , J. Am. Chem. Soc. 2013, 135, 10210. 23805877 10.1021/ja403330m

[36] Q.‐L. Zhu , Q. Xu , Chem. Soc. Rev. 2014, 43, 5468. 24638055 10.1039/c3cs60472a

[37] Z. F. Huang , J. J. Song , K. Li , M. Tahir , Y. T. Wang , L. Pan , L. Wang , X. W. Zhang , J. J. Zou , J. Am. Chem. Soc. 2016, 138, 1359. 26777119 10.1021/jacs.5b11986

[38] M. Hu , J. Reboul , S. Furukawa , L. Radhakrishnan , Y. Zhang , P. Srinivasu , H. Iwai , H. Wang , Y. Nemoto , N. Suzuki , S. Kitagawa , Y. Yamauchi , Chem. Commun. 2011, 47, 8124. 10.1039/c1cc12378e 21691616

[39] S. J. Yang , S. Nam , T. Kim , J. H. Im , H. Jung , J. H. Kang , S. Wi , B. Park , C. R. Park , J. Am. Chem. Soc. 2013, 135, 7394. 23647071 10.1021/ja311550t

[40] W. Xia , R. Q. Zou , L. An , D. G. Xia , S. J. Guo , Energy Environ. Sci. 2015, 8, 568.

[41] M. Eddaoudi , J. Kim , N. Rosi , D. Vodak , J. Wachter , M. O'keeffe , O. M. Yaghi , Science 2002, 295, 469. 11799235 10.1126/science.1067208

[42] P. T. Hsieh , Y. C. Chen , K. S. Kao , C. M. Wang , Appl. Phys. A 2008, 90, 317.

[43] X. Q. Wei , B. Y. Man , M. Liu , C. S. Xue , H. Z. Zhuang , C. J. Yang , Phys. B: Condens. Matter 2007, 388, 145.

[44] H. Tong , S. X. Ouyang , Y. P. Bi , N. Umezawa , M. Oshikiri , J. H. Ye , Adv. Mater. 2012, 24, 229. 21972044 10.1002/adma.201102752

[45] J. Yan , X. S. Fang , L. D. Zhang , Y. Bando , U. K. Gautam , B. Dierre , T. Sekiguchi , D. Golberg , Nano Lett. 2008, 8, 2794. 18687012 10.1021/nl801353c

[46] X. Huang , M.‐G. Willinger , H. Fan , Z.‐L. Xie , L. Wang , A. Klein‐Hoffmann , F. Girgsdies , C.‐S. Lee , X.‐M. Meng , Nanoscale 2014, 6, 8787. 24954555 10.1039/c4nr01575d

[47] J. Zhang , M. Zhang , R. Sun , X. Wang , Angew. Chem., Int. Ed. 2012, 51, 10292.

[48] G. G. Zhang , M. W. Zhang , X. X. Ye , X. Q. Qiu , S. Lin , X. C. Wang , Adv. Mater. 2014, 26, 805. 24170645 10.1002/adma.201303611

[49] Y. Hou , Z. Wen , S. Cui , X. Guo , J. Chen , Adv. Mater. 2013, 25, 6291. 23996281 10.1002/adma.201303116

[50] S. Yang , Y. Gong , J. Zhang , L. Zhan , L. Ma , Z. Fang , R. Vajtai , X. Wang , P. M. Ajayan , Adv. Mater. 2013, 25, 2452. 23450777 10.1002/adma.201204453

[51] Y. Hou , F. Zuo , A. P. Dagg , J. Liu , P. Feng , Adv. Mater. 2014, 26, 5043. 24848321 10.1002/adma.201401032

[52] J. W. Hong , D. H. Wi , S. U. Lee , S. W. Han , J. Am. Chem. Soc. 2016, 138, 15766. 27933998 10.1021/jacs.6b10288

[53] X. Wang , Q. Xu , M. R. Li , S. Shen , X. L. Wang , Y. C. Wang , Z. C. Feng , J. Y. Shi , H. X. Han , C. Li , Angew. Chem., Int. Ed. 2012, 51, 13089. 10.1002/anie.201207554 23161569

[54] X. Zong , H. J. Yan , G. P. Wu , G. J. Ma , F. Y. Wen , L. Wang , C. Li , J. Am. Chem. Soc. 2008, 130, 7176. 18473462 10.1021/ja8007825

[55] G. Kresse , J. Furthmüller , Comput. Mater. Sci. 1996, 6, 15.

[56] G. Kresse , J. Furthmüller , Phys. Rev. B 1996, 54, 11169. 10.1103/physrevb.54.11169 9984901

[57] P. E. Blöchl , Phys. Rev. B 1994, 50, 17953. 10.1103/physrevb.50.17953 9976227

[58] G. Kresse , D. Joubert , Phys. Rev. B 1999, 59, 1758.

[59] J. P. Perdew , K. Burke , M. Ernzerhof , Phys. Rev. Lett. 1996, 77, 3865. 10062328 10.1103/PhysRevLett.77.3865

[60] J. Heyd , G. E. Scuseria , M. Ernzerhof , J. Chem. Phys. 2003, 118, 8207.

[61] H. J. Monkhorst , J. D. Pack , Phys. Rev. B: Solid State 1976, 13, 5188.

