# Supplementary material for: Metal–Organic Framework‐Derived ZnO/ZnS Heteronanostructures for Efficient Visible‐Light‐Driven Photocatalytic Hydrogen Production
Source: Adv Sci (Weinh). 2018 Jan 3;5(4):1700590. doi: 10.1002/advs.201700590 (PMC5908348; doi:10.1002/advs.201700590)
Supplement: Supplementary file 1 — Supplementary [file ADVS-5-1700590-s001.pdf]

Copyright WILEY-VCH Verlag GmbH & Co. KGaA, 69469 Weinheim, Germany, 2018.

ADVANCED SCIENCE
Open Access

Supporting Information

for *Adv. Sci.,* DOI: 10.1002/advs.201700590

Metal–Organic Framework-Derived ZnO/ZnS Heteronanostructures for Efficient Visible-Light-Driven Photocatalytic Hydrogen Production

Xiuxia Zhao, Jianrui Feng, Jingwei Liu, Jia Lu, Wei Shi, Guangming Yang, Guichang Wang, Pingyun Feng,* and Peng Cheng**

Copyright WILEY-VCH Verlag GmbH & Co. KGaA, 69469 Weinheim, Germany, 2016.

Supporting Information

Metal-Organic Framework-Derived ZnO/ZnS Heteronanostructures for Efficient Visible-Light-Driven Photocatalytic Hydrogen Production

Xiuxia Zhao, Jianrui Feng, Jingwei Liu, Jia Lu, Wei Shi, Guangming Yang, Guichang Wang, Pingyun Feng,* and Peng Cheng**

Table of Contents

**Figure S1**	SEM image of the as-prepared MOF-5	S3
**Figure S2**	PXRD patterns of the as-prepared MOF-5	S4
**Figure S3**	TG curve of the as-prepared MOF-5	S5
**Figure S4**	PXRD patterns of the rough ZnS@C particles before calcination process	S6
**Figure S5**	SEM image of the rough ZnS@C particles before calcination process	S7
**Figure S6**	The particle size distribution of ZnS@C	S8
**Figure S7**	TG curve of ZnOS@C composite	S9
**Figure S8**	XRD patterns of ZnS@C calcined at different temperatures in air	S10
**Figure S9**	TG curves of ZnOS-n composites	S11
**Figure S10**	EDS spectra of the ZnOS-n	S12
**Figure S11**	XPS S 2p spectra of ZnOS-n	S13
**Figure S12**	XPS Zn 2p spectra of ZnOS-n	S14
**Figure S13**	XPS C 1s spectra of ZnOS-n	S15
**Figure S14**	The particle size distributions of ZnOS-n	S16

**Figure S15**	The pore-size distributios of ZnOS-n	S17
**Figure S16**	UV-visible absorption spectra of ZnS@C calcined at different temperatures in air	S18
**Figure S17**	Comparison of photocatalytic HER activity of ZnS@C calcined at different temperatures in air	S19
**Figure S18**	Calculated band structure plot of ZnS and ZnO	S20
**Figure S19**	XRD patterns of ZnOS-30 after photocatalysis for eight runs and after retreatment	S21
**Table 1**	The content ratio of ZnO and ZnS in ZnOS-n	S22
**Table 2**	Carbon content of ZnOS-n from element analysis	S23
**Table 3**	Summary of the photocatalytic H_2 evolution on ZnO/ZnS materials	S24
**Reference**		S24

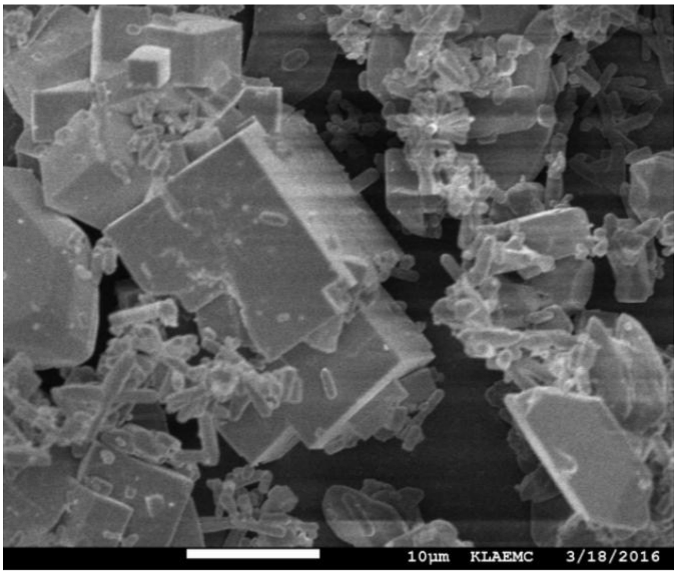

Figure S1. SEM image of the as-prepared MOF-5.

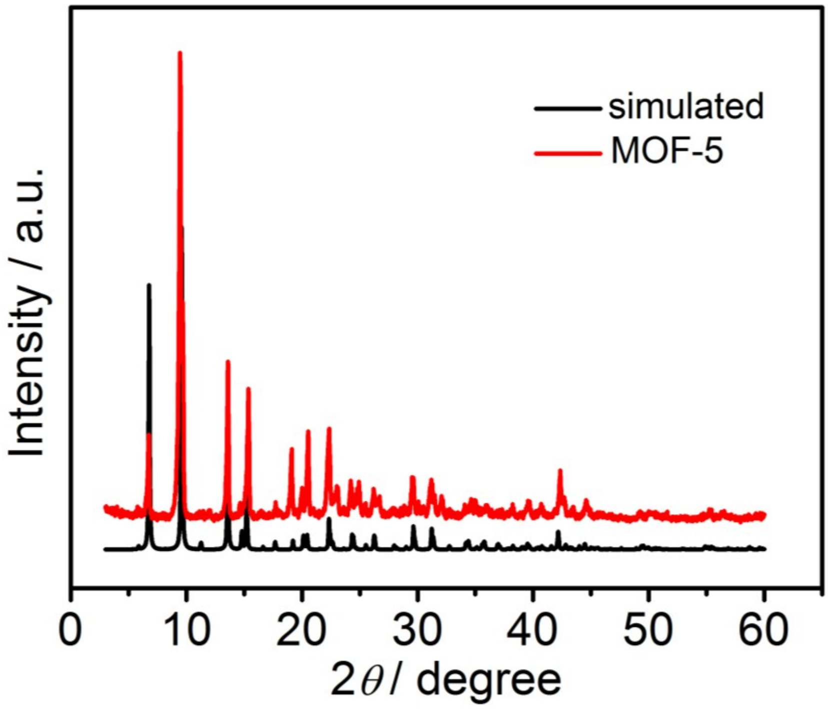

Figure S2 Experimental and simulated PXRD patterns of MOF-5.

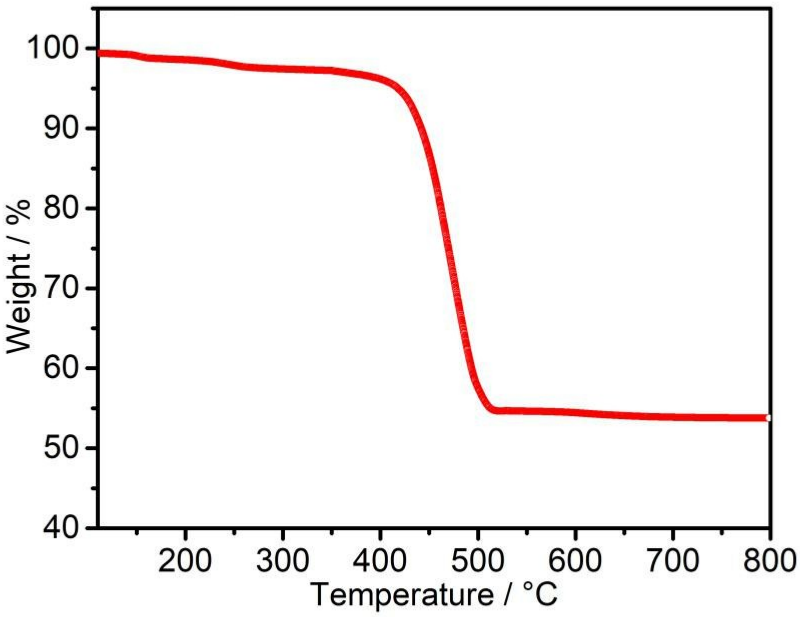

Figure S3. TG curve of the as-prepared MOF-5.

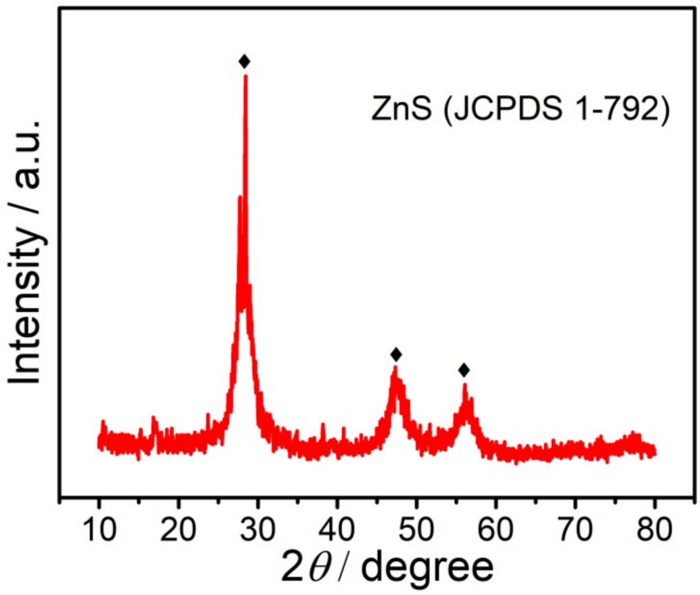

Figure S4 PXRD patterns of the rough ZnS@C particles before calcination.

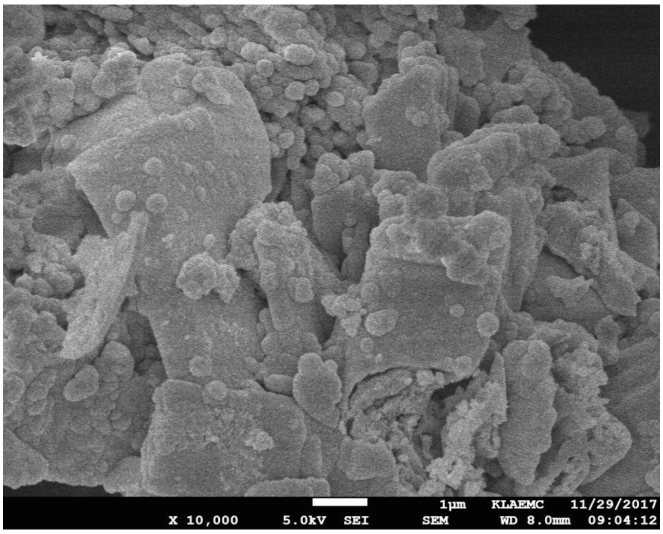

Figure S5. SEM image of the rough ZnS@C particles before calcination.

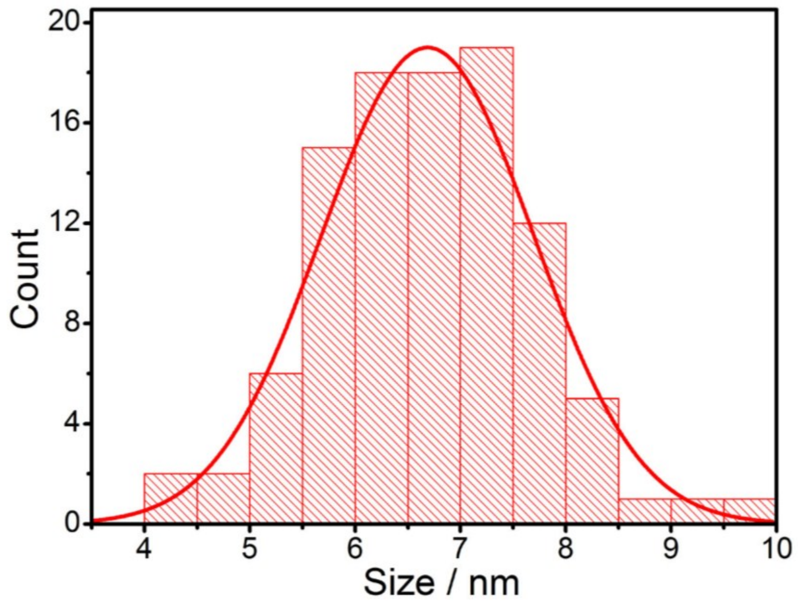

Figure S6 The particle size distribution of ZnS@C. The sampling number is 100. The centered particle size is ~ 7 nm.

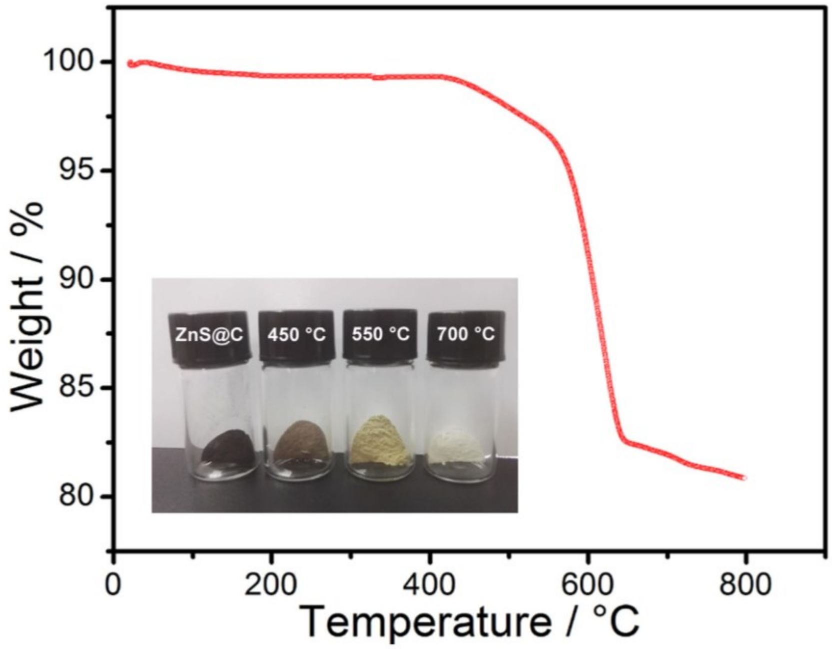

Figure S7 TG curve of ZnS@C composite in air, color photograph (inset) of ZnS@C and the calcined ZnS@C at 450 °C, 550 °C and 700 °C in air for 30 min.

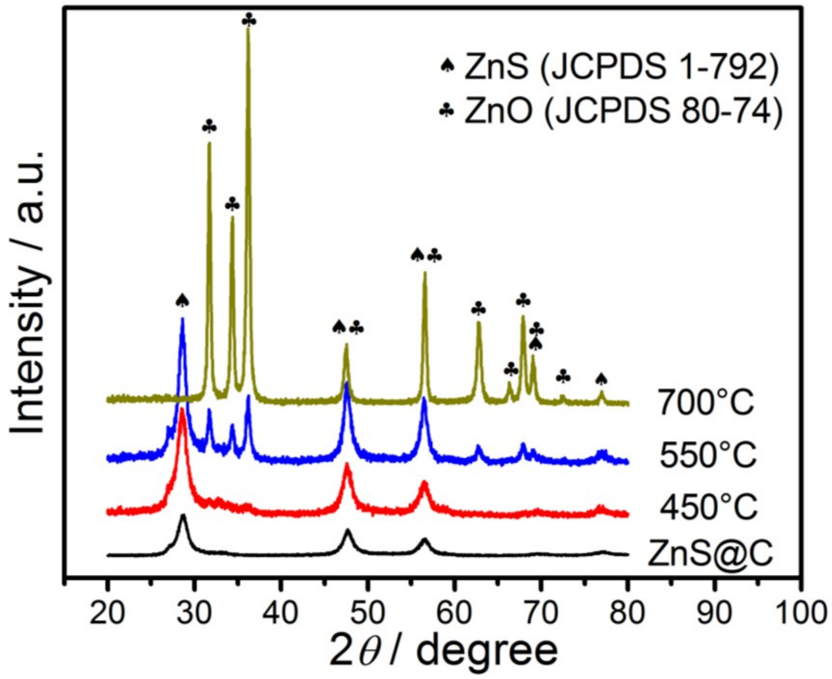

Figure S8 PXRD patterns of ZnS@C and the calcined ZnS@C at 450 °C, 550 °C and 700 °C in air for 30 min.

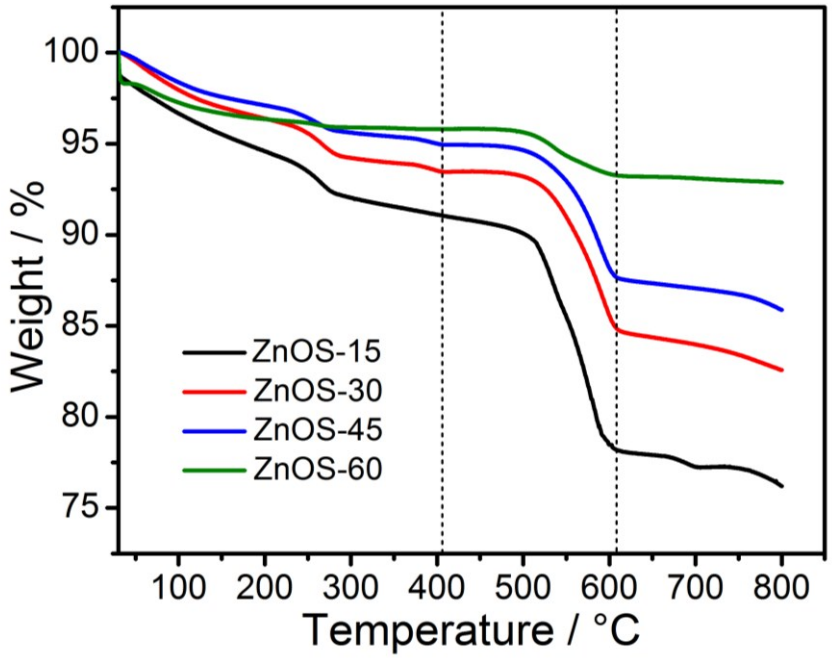

Figure S9 TGA curves of ZnOS-n in air. The stage (40-410 °C) is attributed to the removal of adsorbed water and carbon residue. The stage of 410-612 °C has a significant weight loss, which is mainly derived from the transformation from ZnS to ZnO of ZnO/ZnS heterostructures.

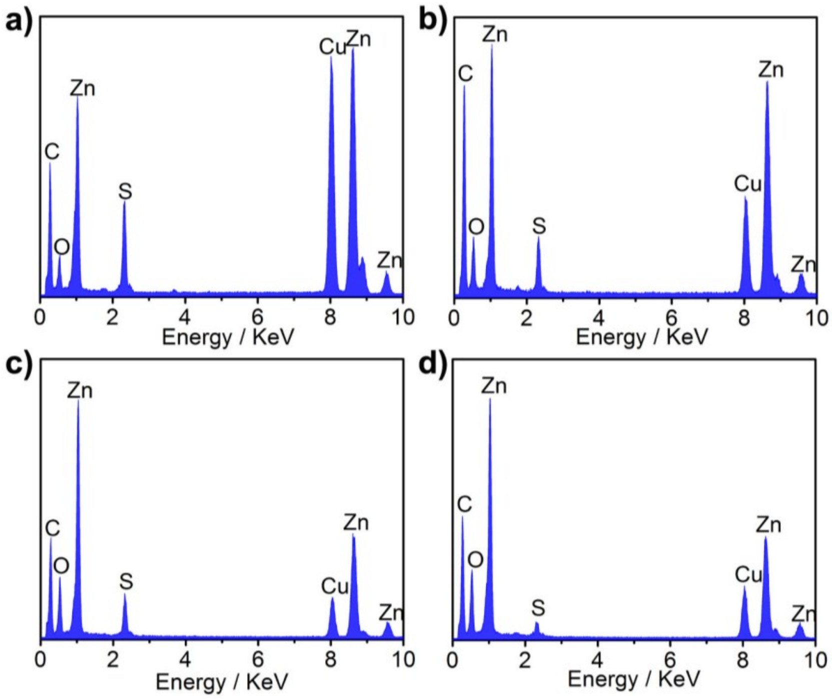

Figure S10 EDS spectra of ZnOS-15 (a), ZnOS-30 (b), ZnOS-45 (c), ZnOS-60 (d).

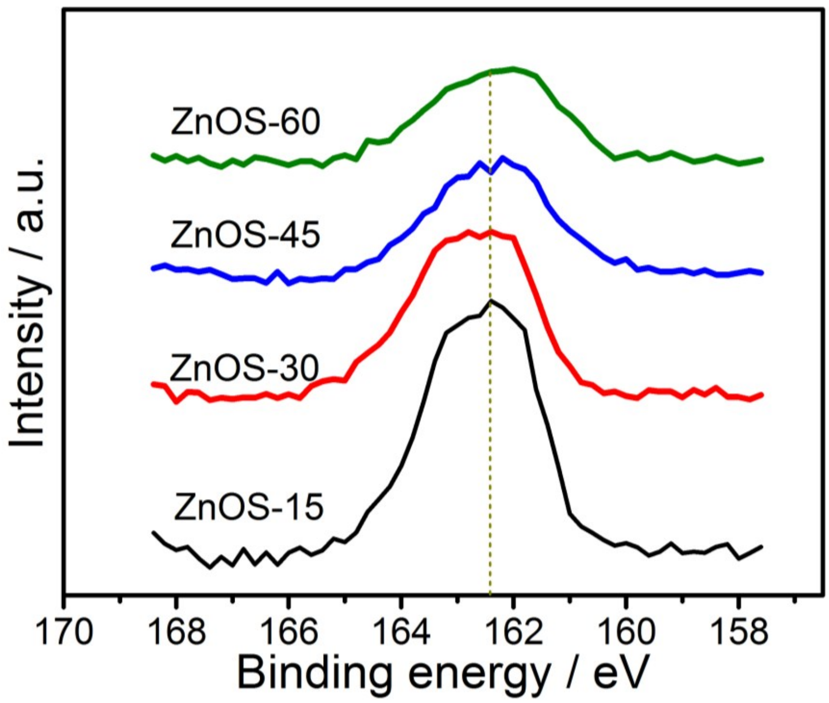

Figure S11 XPS survey spectra with the corresponding S 2p spectra of ZnOS-n.

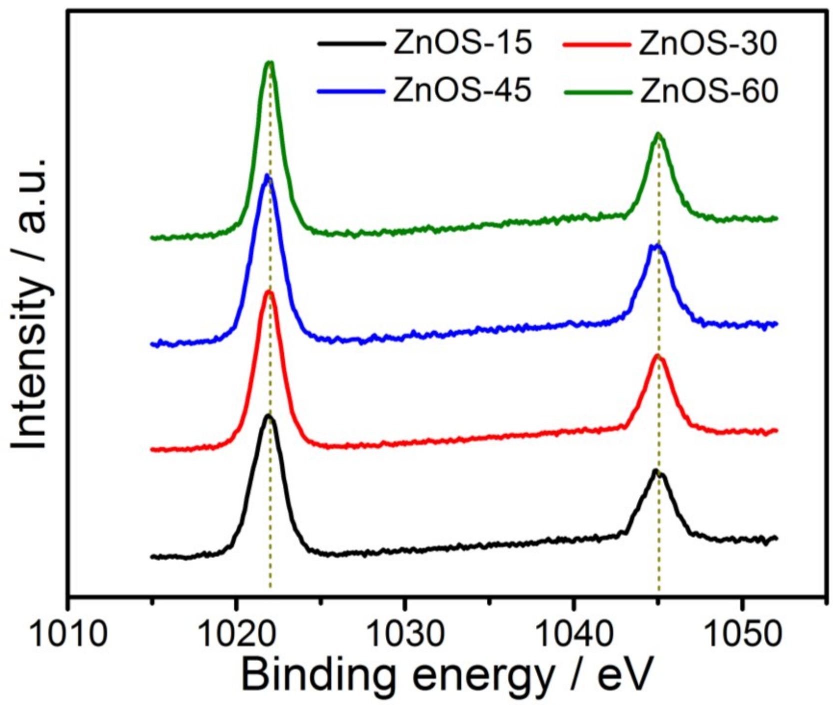

Figure S12 XPS survey spectra with the corresponding Zn 2p spectra of ZnOS-n.

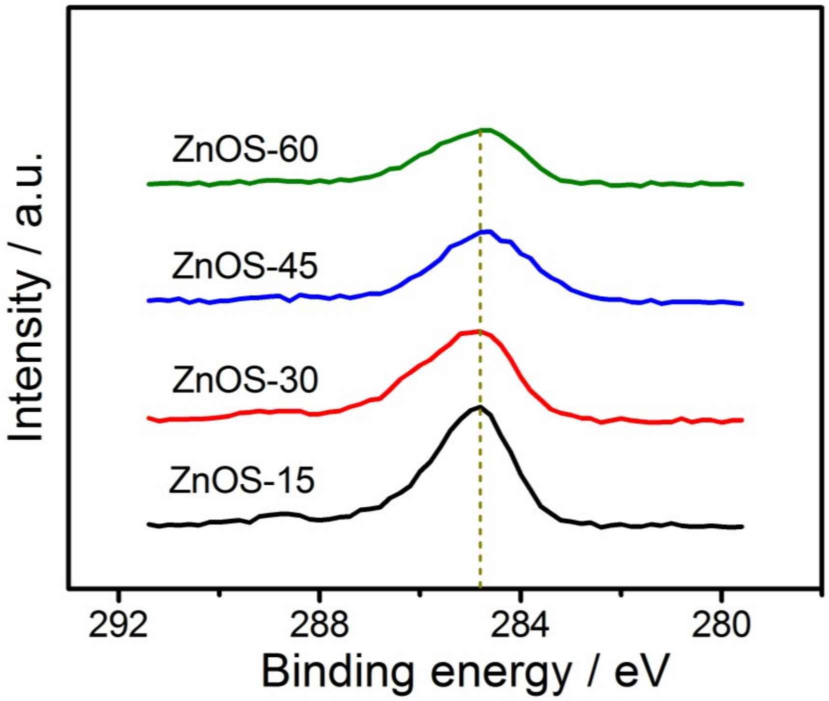

Figure S13 XPS spectra with the corresponding C 1s spectra of ZnOS-n.

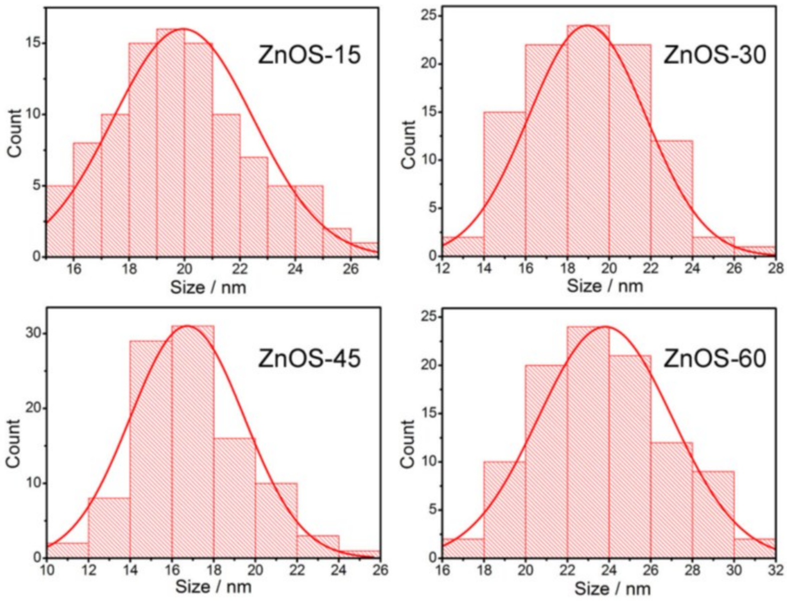

Figure S14 The particle size distributions of ZnOS-n. Their sampling numbers are 100, respectively.

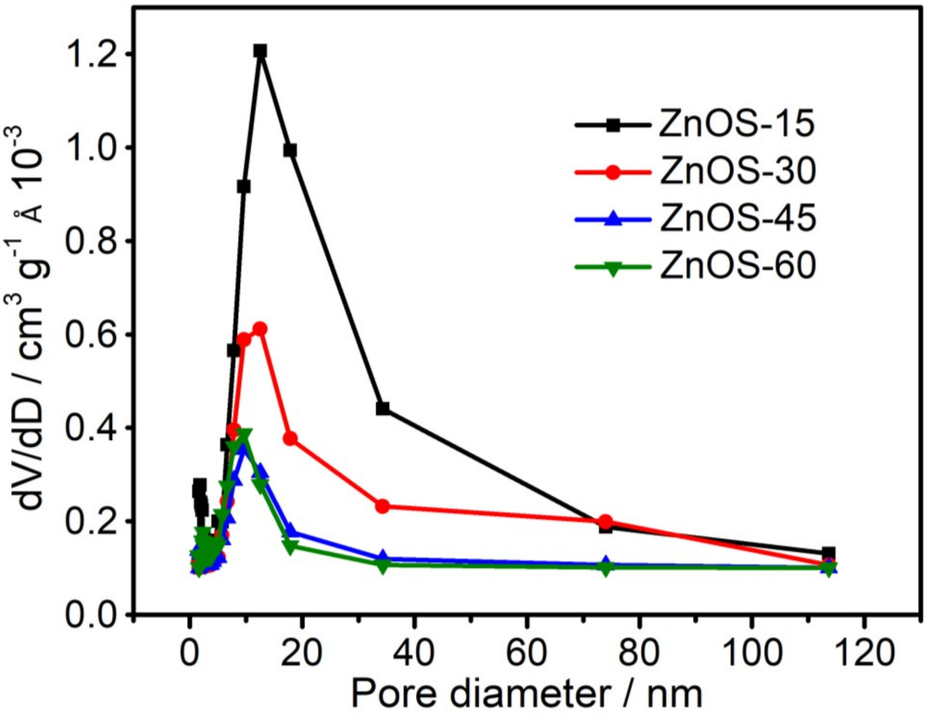

Figure S15 The pore-size distributions of ZnOS-n.

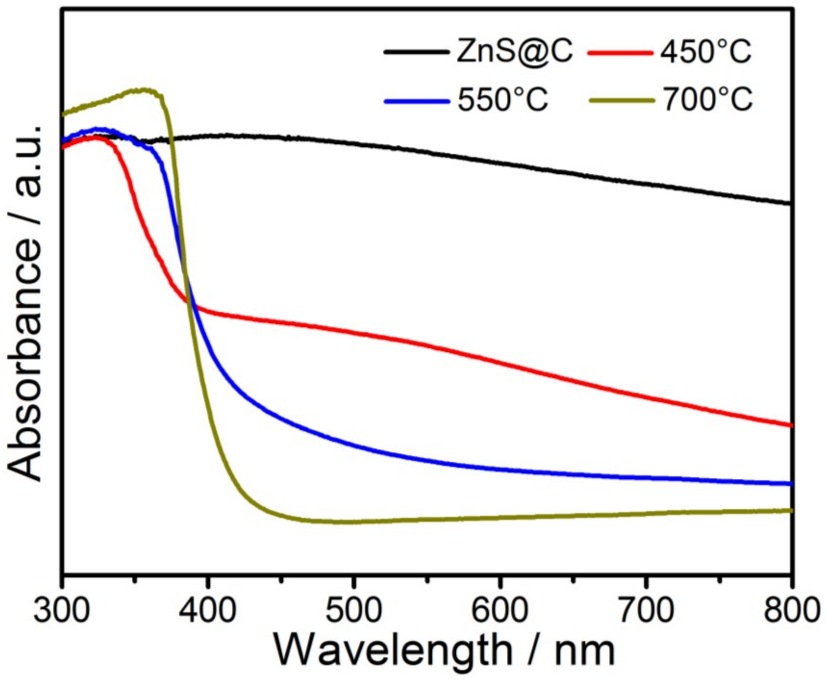

Figure S16 UV-visible absorption spectra of ZnS@C and the calcined ZnS@C at 450 °C, 550 °C and 700 °C in air for 30 min.

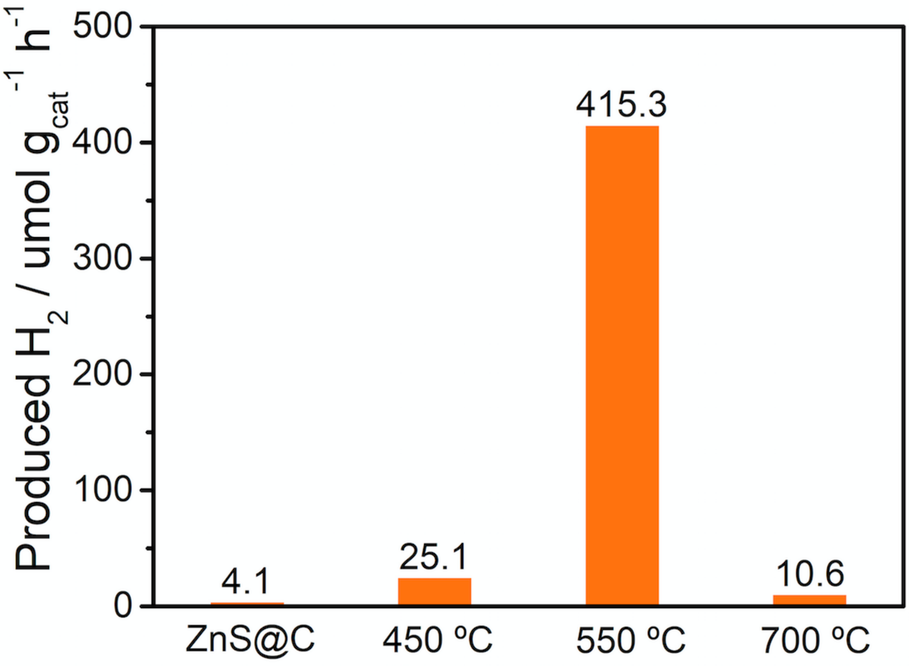

Figure S17 Comparison of tje photocatalytic HER activity of ZnS@C and the calcined ZnS@C at 450 °C, 550 °C and 700 °C in air for 30 min.

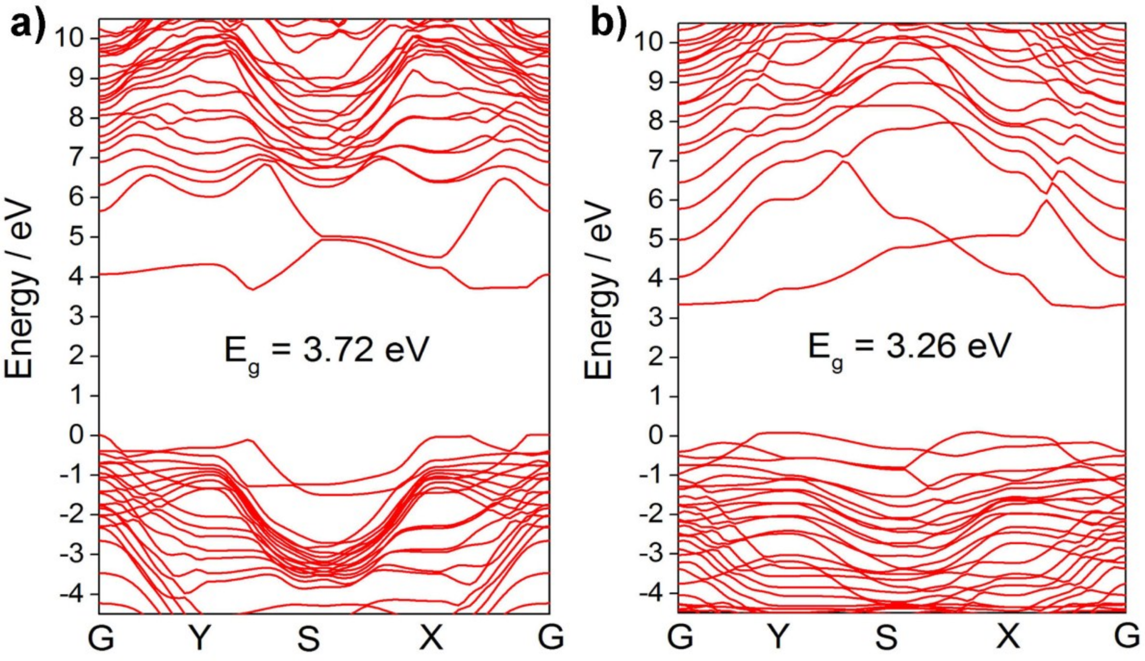

Figure S18 Calculated band structure plot of ZnS (a) and ZnO (b).

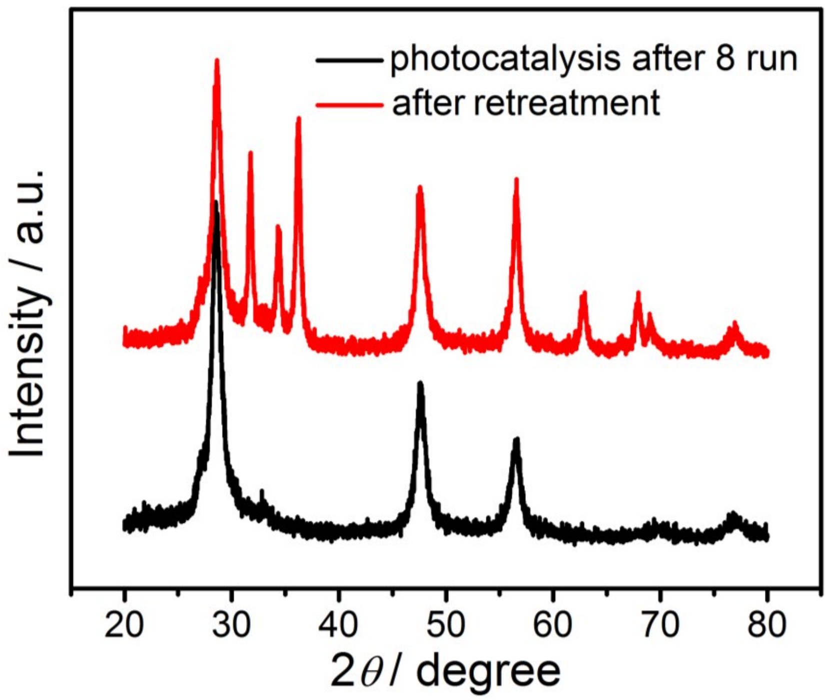

Figure S19 XRD patterns of ZnOS-30 after photocatalysis for 8 runs and after retreatment.

Table S1 The content ratios of ZnO and ZnS in ZnOS-n.

	The molar ratio of ZnS and ZnO (n_{ZnS}/n_{ZnO})		
	TG	EDS	XPS
ZnOS-15	5.06	4.21	4.96
ZnOS-30	1.08	1.12	1.10
ZnOS-45	0.70	0.55	0.76
ZnOS-60	0.16	0.09	0.14

Table S2 Carbon content of the heterostructures ZnOS-n from element analysis.

	ZnOS-15	ZnOS-30	ZnOS-45	ZnOS-60
Carbon content (wt%)	0.27	0.25	0.22	0.16

Table S3 Summary of the photocatalytic H_2 evolution on ZnO/ZnS materials.

Photocatalyst	Light source	Sacrificial agent	Cocatalyst	Activity $\mu mol h^{-1} g^{-1}$	Stability	Ref
1D ZnO/ZnS	(350 W Xe) visible light	glycerol	none	384	12h	[1]
ZnO/ZnS nanorods	125 W Hg UV	glycerol	none	2608.7	1h	[2]
ZnO/ZnS nanorods	solar-simulated light (500 W Xe)	glycerol	none	388.4	1h	[2]
ZnS/ZnO heterostructure	150 W Xe lamp	SO_3^{2-}	none	494.8	<7.5h	[3]
ZnS/ZnO nanocomposite	(300 W Xe) >400 nm	S^{2-}/SO_3^{2-}	none	187	16h	[4]
ZnO@ZnS nanorods	UV irradiation	$S^{2-}/SO_3^{2-}/Cl^-$	none	5310	3h	[5]
Ag_2S-coupled ZnO@ZnS	visible Light	$S^{2-}/SO_3^{2-}/Cl^-$	none	168	15h	[5]
ZnO/ZnS heterostructure	(300 W Xe) >420 nm	S^{2-}/SO_3^{2-}	none	415.3	>80h	This work

Referenece

[1] D. Bao, P. Gao, X. Y. Zhu, S. C. Sun, Y. Wang, X. B. Li, Y. J. Chen, H. Zhou, Y. B. Wang, P. P. Yang, *Chem. Eur.J.* **2015**, *21,* 12728.

[2] H. X. Sang, X. T. Wang, C. C. Fan, F. Wang, *Int J Hydrogen Energy* **2012**, *37*, 1348.

[3] E. Hong, J. H. Kim, *Int J Hydrogen Energy* **2014**, *39*, 9985.

[4] H. Zhao, Y. M. Dong, P. P. Jiang, X. M. Wu, R. X. Wu, Y. M. Chen, *RSC Adv.* **2015**, *5,* 6494.

[5] M.-H. Hsu, C.-J. Chang, H.-T. Weng, *ACS Sustainable Chem. Eng.* **2016**, *4*, 1381.
